# Impact of dual-layer spectral CT-derived stopping power ratio on single-energy CT-based carbon-ion treatment planning for thoracic cancer

**DOI:** 10.1371/journal.pone.0358184

**Published:** 2026-09-15

**Authors:** Euntaek Yoon, Sung Hyun Lee, Hyeongmin Jin, Tae Ho Jang, Sungjin Yoon, Hyung Jin Choun, Soon Ho Yoon, Jin Mo Goo, Jong Min Park

**Affiliations:** 1 Department of Radiation Oncology, Seoul National University Hospital, Seoul, Republic of Korea; 2 Interdisciplinary Program, Bioengineering Major, Graduate School, Seoul National University, Seoul, Republic of Korea; 3 Institute of Radiation Medicine, Seoul National University Medical Research Center, Seoul, Republic of Korea; 4 Biomedical Research Institute, Seoul National University Hospital, Seoul, Republic of Korea; 5 Project Group of the Gijang Heavy Ion Medical Accelerator, Seoul National University Hospital, Seoul, Republic of Korea; 6 Department of Radiation Oncology, Seoul National University College of Medicine, Seoul, Republic of Korea; 7 Department of Radiology, National Jewish Health, Denver, Colorado, United States of America; 8 Department of Radiology, Seoul National University College of Medicine, Seoul National University Hospital, Seoul, Republic of Korea; Chung-Ang University Gwangmyeong Hospital, KOREA, REPUBLIC OF

## Abstract

**Purpose:**

To evaluate the impact of stopping-power ratio (SPR) predictions derived from dual-layer detector-based spectral CT (DLCT) on conventional single-energy CT (SECT)-based carbon-ion treatment planning for thoracic cancer.

**Methods:**

Twenty patients with thoracic cancer who underwent non-contrast DLCT were retrospectively included. DLCT-based SPR images were generated from effective atomic number and relative electron density maps using the Bethe equation. SECT-based SPR was derived using a Hounsfield unit look-up table generated via stoichiometric calibration of a commercial electron density phantom. Using the same phantom, a phantom-based prediction assessment was performed by comparing SECT- and DLCT-based SPR predictions with theoretical SPR values for the inserts. For patient analysis, normal-tissue SPR values derived from SECT and DLCT were compared. Carbon-ion treatment plans were first generated based on SECT images and then recalculated using DLCT-derived SPR images. Range differences in the beam’s-eye view and dose–volume histogram parameters for the planning target volume and organs at risk were evaluated.

**Results:**

In the phantom-based assessment, the root-mean-square error relative to the theoretical SPR was 5.57% for SECT and 2.83% for DLCT. In patient images, mean SPR differences between SECT and DLCT were 3.62% for the lungs, 1.93% for the esophagus, 0.04% for the heart, 0.93% for the spinal cord, and 1.53% for the ribs. Across the patient cohort, the mean recalculated range difference was 0.71 ± 0.89 mm, corresponding to a relative difference of 0.60 ± 0.67%. No statistically significant differences were observed in target dose metrics, whereas selected organ-at-risk metrics showed statistically significant differences between SECT-based plans and DLCT-based recalculations.

**Conclusion:**

DLCT-derived SPR prediction showed better agreement with theoretical phantom SPR values in the phantom-based prediction assessment and produced tissue-dependent SPR differences in thoracic patient images compared with conventional SECT-based prediction. Recalculation on DLCT-derived SPR maps resulted in modest but systematic range differences and limited changes in selected dose metrics. These results may inform the implementation and future independent validation of patient-specific DLCT-based SPR workflows in carbon-ion treatment planning for thoracic cancer.

## 1. Introduction

Carbon-ion radiotherapy (CIRT) centers have been steadily increasing worldwide [1]. The CIRT modality has witnessed rapid clinical adoption because heavy-ion beams offer superior physical and biological advantages over conventional photon therapy. Like protons, carbon-ion beams exhibit a characteristic Bragg peak, which enables conformal dose delivery to the target while sparing surrounding normal tissues. However, the steep dose fall-off makes heavy-ion therapy highly sensitive to range uncertainties along the beam path Consequently, larger planning target volume (PTV) margins are required, which can compromise these dosimetric advantages.[2,3]. Stopping power ratio (SPR) estimation from computed tomography (CT) images is one of the principal contributors to particle-beam range uncertainty. In proton therapy, an uncertainty of approximately 3.5% has been used to account for CT-number accuracy and conversion to proton relative stopping power [4,5].

Currently, CIRT is generally performed using single-energy CT (SECT). In many centers, CT number-to-SPR conversion is conducted using a Hounsfield unit look-up table (HLUT), which is generated from theoretical CT numbers for human tissues obtained by stoichiometric calibration [6,7]. Because both the CT number and SPR are strongly influenced by the electron density of tissues, this approach is reasonable and has been widely adopted in clinical practice. However, it remains heuristic and cannot fully account for tissue-specific variations in the elemental composition. Tissues with the same CT number may have different SPR values, and conversely, tissues with different CT numbers may have similar SPR values.

Dual-energy CT (DECT) enables voxel-wise SPR estimation by deriving the effective atomic number (EAN) and relative electron density (RED) information from two image sets acquired using different X-ray spectra. Several phantom studies using tissue-equivalent inserts with known reference values have shown that DECT-based SPR estimation is more accurate than SECT-based estimation [8–10]. More recently, studies have investigated the extent to which DECT-based treatment planning can reduce uncertainty compared with conventional SECT-based planning, reporting reductions in range uncertainty of approximately 1.5%.[11] Various DECT acquisition techniques, including dual-source CT and rapid kV switching, as well as different methods for deriving EAN and RED from DECT images, have been proposed [10,12,13]. Among these techniques, dual-layer detector-based spectral CT (DLCT) has several practical advantages, including reduced motion-related misregistration owing to simultaneous acquisition, retrospective spectral analysis from routine CT scans, and user-friendly generation of EAN and RED maps using vendor-provided algorithms.

In thoracic cancer treatment, CIRT is used for early-stage inoperable non-small-cell lung cancer, locally advanced or recurrent tumors resistant to conventional radiotherapy, and reirradiation cases. It has also been explored for rare thoracic malignancies, such as thymic carcinoma and pleural mesothelioma, for which high precision and high linear energy transfer may be beneficial. Previous patient studies have applied DECT-based SPR prediction to head and brain tumors as well as head-and-neck cancer cases, comparing SECT- and DECT-derived images in terms of particle range and treatment-plan differences [14–17]. Compared with the treatment of brain and head-and-neck tumors, thoracic particle therapy involves additional anatomical and motion-related uncertainties, and the specific impact of DLCT-derived SPR information on carbon-ion treatment planning for thoracic cancer remains insufficiently studied [18,19]. Specifically, no study has yet investigated the range and dosimetric impact of recalculating SECT-based thoracic carbon-ion treatment plans using DLCT-derived SPR maps.

In this study, we investigated the potential impact of DLCT-derived SPR information on conventional SECT-based carbon-ion treatment planning for patients with thoracic cancer. First, a phantom-based prediction assessment was performed by comparing SECT- and DLCT-derived SPR values with theoretical values for CIRS phantom inserts. SECT-based treatment plans were then generated using patient CT images and recalculated on DLCT-derived SPR maps to evaluate differences in beam range and dosimetric parameters.

## 2. Materials and Methods

### 2.1. Dual-layer spectral CT system

All the imaging was performed using a dual-layer spectral CT system (IQon Spectral CT, Philips Healthcare, Best, The Netherlands). The system uses a single X-ray source and two vertically stacked scintillator-detector layers that can simultaneously acquire low- and high-energy spectral details from the same projection geometry. The acquired spectral data are decomposed into Compton-scatter-like and photoelectric-like basis components [20–23]. In the Philips Iqon workflow, these spectral basis data are stored as a spectral base image (SBI), a reconstructed DICOM spectral dataset from which retrospective spectral results can be generated, including virtual monochromatic images, EAN maps, and RED maps [24]. In this study, the conventional polychromatic Hounsfield unit (HU) image reconstructed from DLCT data was used as the SECT-equivalent input for HLUT-based SPR calculation [25]. For the DLCT-based SPR prediction, vendor-generated EAN and RED maps derived from the SBI were used.

### 2.2. Patient selection and acquisition settings

We retrospectively included 20 consecutive patients with thoracic cancer who underwent diagnostic non-contrast chest DLCT between 1 August 2024 and 31 August 2025 and were treated with intensity-modulated radiotherapy at Seoul National University Hospital. This study was approved by the Institutional Review Board of Seoul National University Hospital (IRB No. H-2509-147-1679), which waived the requirement for informed consent. The data were accessed for research purposes from 1 October 2025 to 31 October 2025. The author responsible for data collection had access to identifiable information during data collection, but all data were anonymized before analysis. Patient staging and clinical details are provided in S1 File. Only patients scanned with the institutional non-contrast thoracic protocol (Thorax #1) were included. The acquisition and reconstruction parameters are summarized in Table 1 (first row). To generate the SECT-based calibration curve, an electron density phantom (CIRS Model 062M; CIRS, Inc., Norfolk, VA, USA) was scanned using a separate thoracic phantom protocol (Thorax #2), which was similar to the patient protocol (Thorax #1) but used a different tube current–time product and slice thickness, as listed in Table 1 (second row).

**Table 1:** Scanning and reconstruction parameters for patient and phantom CT acquisitions using the dual-layer detector-based spectral CT (DLCT) system.

Protocol	Tube voltage (kVp)	Tube current time (mAs)	Collimation (mm)	Rotation time (s)	Pitch (mm)	Slice thickness (mm)	Reconstruction filter	Spectral level
Thorax #1	120	56	64 × 0.625	0.33	1.015	3	YC	4
Thorax #2	120	75	64 × 0.625	0.33	1.015	1	YC	4

### 2.3. DLCT-based SPR prediction

DLCT-based SPR prediction was performed using vendor-generated voxel-wise EAN and RED maps derived from the SBI. Unlike the HLUT-based SPR prediction in the SECT workflow, the DLCT-based SPR values can be calculated directly from EAN and RED maps using the Bethe equation (Eq. 1).


$$\begin{array}{c} SPR=RED\frac{\text{ln}\left(\frac{\text{2}m_{e}c^{\text{2}}\beta^{\text{2}}}{I_{m}\left(\text{1}-\beta^{\text{2}}\right)}\right)-\beta^{\text{2}}}{\text{ln}\left(\frac{\text{2}m_{e}c^{\text{2}}\beta^{\text{2}}}{I_{w}\left(\text{1}-\beta^{\text{2}}\right)}\right)-\beta^{\text{2}}} \end{array}$$ (1)

**Fig 1:**
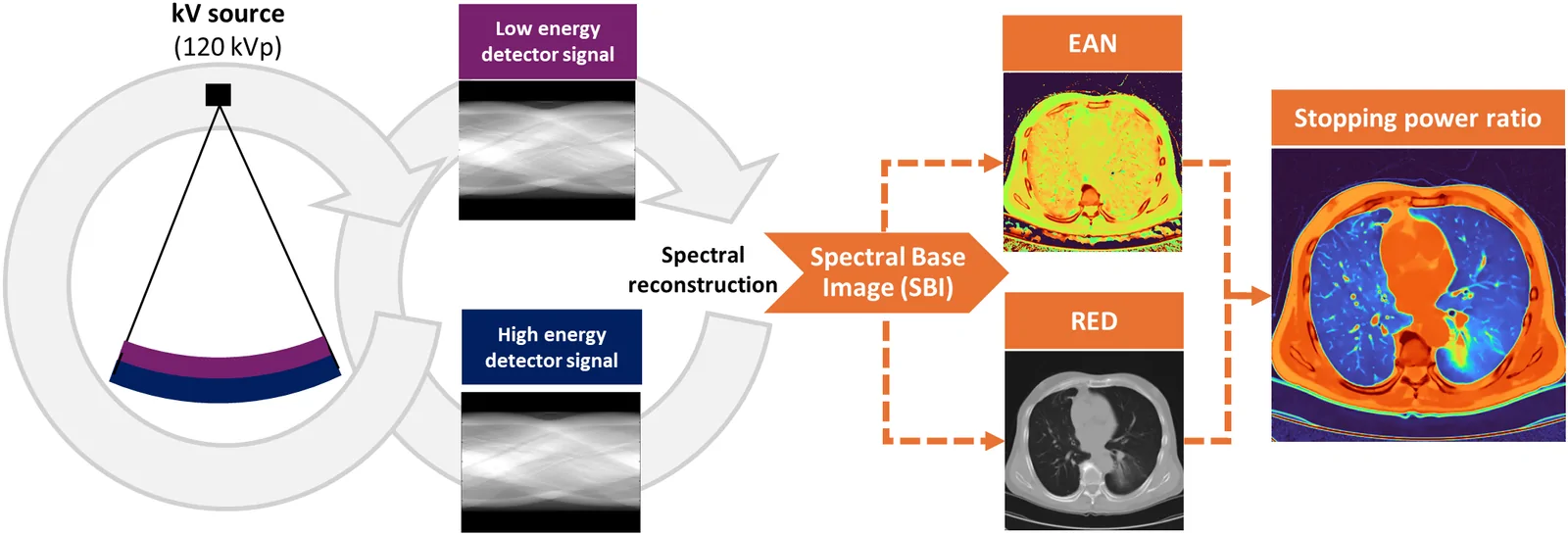
Schematic for predicting the stopping power ratio (SPR) using DLCT. After a single DLCT acquisition, the spectral data are stored as a spectral base image (SBI) for retrospective spectral analysis. Effective atomic number (EAN) and relative electron density (RED) images are generated from the SBI using the Philips IQon workflow. The EAN map is converted to a voxel-wise mean excitation energy map, which is combined with the RED map to calculate the DLCT-based SPR map using the Bethe equation.

Here, *m*_*e*_ denotes the electron mass; *β* is the particle-beam velocity relative to the speed of light *c*; *I*_*m*_ and *I*_*w*_ are the mean excitation energies of the medium and water, respectively. For *β*, a carbon-ion beam energy of 131 MeV/u was assumed [26]. The mean excitation energy of water was set to 75.3 eV [6,27]. The voxel-wise mean excitation energy of the medium was estimated from the EAN image by applying a fifth-order polynomial fit to the relationship between the EAN and the natural logarithm of the mean excitation energy (ln *I*), which was generated using human reference tissue information [28]. An exponent of 2.94 was used to derive EAN from the material-specific elemental composition weighted by the fraction of electrons associated with each element [25]. The corresponding EAN-to-ln *I* conversion function is provided in S1 File. The resulting mean excitation energy map was then combined with the RED image to generate the DLCT-based SPR map according to Eq. (1). Fig 1 shows the overall DLCT-based SPR prediction workflow.

For SECT-based SPR prediction, an HLUT was generated using stoichiometric calibration [7,29]. Its construction followed a previously reported consensus guide, including the definition of tissue groups and the connection of corresponding tissue-specific line segments [6].

### 2.4. Phantom-based assessment of SPR prediction agreement

Using the plugs of the CIRS electron density phantom, we assessed the agreement of the SECT- and DLCT-based SPR predictions with theoretical SPR values. For the DLCT-based SPR prediction, EAN and RED maps of the phantom were generated from the DLCT dataset, and SPR maps were generated using the method described in Section 2.3. For each insert, the mean SPR value was obtained from three consecutive slices centered on the insert and used as the representative DLCT-based SPR (SPR_DLCT_). To reduce partial-volume effects, the region of interest (ROI) was defined as 75% of the plug radius.

For SECT-based SPR prediction, the mean HU value of each insert was obtained using the same slice-selection and ROI scheme, and the corresponding SECT-based SPR (SPR_SECT_) was derived using the HLUT described in Section 2.3. Because two inserts were available for each material, the mean HU value of each individual insert was used as the representative value and applied to the HLUT. The final representative SPR value for each material was then determined by averaging the SPR values derived from the two inserts.

The theoretical SPR of each plug (SPR_theor_) was calculated using Eq. (1) and the Bragg additivity rule, based on the plug mass density provided by the manufacturer and the elemental compositions reported in the literature [10]. These SPR_theor_ values were used as calculated reference values and depend on the assumed elemental compositions and mean excitation energies rather than representing an absolute ground truth. The inserts used in this analysis and their elemental compositions are provided in S1 File. The agreement of each method with the theoretical values was quantified by calculating the relative residuals of SPR_SECT_ and SPR_DLCT_ with respect to SPR_theor_ as follows:


$$\begin{array}{c} \frac{\left(SP{\text{R}}_{me\text{thod}}-SP{\text{R}}_{\mathrm{th}eor}\right)}{SP{\text{R}}_{\mathrm{th}eor}}\times 100\% \end{array}$$ (2)

where SPR_method_ represents either SPR_SECT_ or SPR_DLCT_.

It should be noted that the CIRS 062M phantom used for this assessment was also used to derive the stoichiometric parameters for the SECT HLUT. Therefore, this insert-based comparison represents a phantom-based prediction assessment and does not constitute an independent validation of DLCT-derived SPR accuracy.

### 2.5. Carbon-ion treatment planning

To generate clinically realistic radiotherapy (RT) plans, the clinical target volume (CTV) and organ-at-risk (OAR) contours for the lungs, heart, esophagus, and spinal cord delineated on the photon RT images were deformably propagated to the DLCT images using MIM Maestro (v7.3.7; MIM Software Inc., Cleveland, OH, USA). The propagated contours were manually corrected and reviewed by a radiation oncologist, after which the planning target volume (PTV) was generated by applying a 5 mm margin to the CTV. The ribs were additionally delineated on the DLCT images using the auto-contouring function implemented in RayStation.

For patients scanned during follow-up, the deformed target contour overlapped with lung tissue and therefore did not represent the physical properties of the original tumor. Gross tumor was identifiable on the DLCT images in 3 of the 20 patients, and the SPR values within these visible tumor regions were close to 1.0. Based on this observation, water-equivalent material properties were assigned to the target region to avoid representing the original tumor as lung tissue in the SPR calculation. Because GTV contours were not consistently available in the original treatment plans, the water-equivalent assignment was applied to the entire CTV in all patients to maintain a consistent approach across the cohort. The overridden CTV volume ranged from 0.80 to 122.76 cm^3^, with a median of 5.84 cm^3^; individual CTV volumes are provided in S1 File. No sensitivity analysis comparing calculations with and without the water-equivalent assignment was performed. OAR dose constraints were defined according to previous studies [30,31].

Carbon-ion treatment planning was performed using the RayStation treatment planning system (v17.0.101.0; RaySearch Laboratories AB, Stockholm, Sweden). Dose calculations were performed with a 2 mm grid size using the pencil beam algorithm (v7.3). RBE-weighted dose was calculated in Gy(RBE) using the modified microdosimetric kinetic model implemented in RayStation [32]. A prescription dose of 60 Gy (RBE) delivered in four fractions was used, based on a previous study [33]. Each fraction was delivered using two ports per day, with a total of four beams. Multifield sequential optimization was performed, and the plans were normalized so that the median dose to the PTV matched the prescription dose.

### 2.6. Comparative analysis of SPR prediction, range, and dosimetric differences

#### 2.6.1. Comparison of SPR prediction between DLCT and SECT for normal tissues.

The SPR values derived from DLCT and SECT were evaluated and compared for normal tissues. Mean SPR was calculated over all voxels within the lungs, spinal cord, heart, esophagus, and ribs delineated in Section 2.5. For each of the 20 patients, the patient-wise relative difference in the tissue mean SPR between SECT and DLCT was evaluated. The relative difference in the mean SPR was calculated as (SPR_SECT_−SPR_DLCT_)/SPR_SECT_. In addition to the ROI-based analysis, representative relative difference maps were examined to qualitatively assess differences in SPR prediction.

#### 2.6.2. Evaluation of range differences via plan recalculation.

For each patient, the SECT image and the corresponding DLCT-based SPR images were imported into RayStation. Carbon-ion treatment plans were first generated on the SECT image following the procedure described in Section 2.5. The resulting plans were then recalculated on the DLCT-based SPR image using the plan recalculation function in RayStation. For both the original SECT-based plans and the DLCT-recalculated plans, range assessment was performed. For each beam direction, 15 line doses were extracted, yielding a total of 60 line doses per patient. Detailed information on the line dose export is provided in S1 File. The range was defined as the point at which the dose reduced to 80% of the prescribed dose on the distal side of the spread-out Bragg peak along the incident beam direction. The range difference distributions between the SECT-based plans and the DLCT-recalculated plans were calculated for each patient and presented using box-and-whisker plots.

#### 2.6.3. Dosimetric comparison between SECT plans and DLCT recalculations.

For the SECT-based plans and the corresponding DLCT recalculations described in Section 2.6.2, target dosimetric parameters were evaluated and compared using dose–volume histograms (DVHs). For the planning target volume (PTV), *D*_98_, *D*_95_, *D*_2_, and mean dose were assessed, where *D*_x_ was defined as the dose covering x% of the target volume. The homogeneity index (HI) was calculated according to ICRU Report 83 as HI = (D_2_ − D_98_)/D_50_, where D_2_, D_98_, and D_50_ represent the doses received by 2%, 98%, and 50% of the PTV, respectively [34]. The conformity index (CI) was defined as the ratio of the target volume covered by the 95% prescription isodose to the total volume enclosed by the 95% prescription isodose [35]. For each DVH parameter, paired values from the SECT-based plan (DVH_SECT_) and DLCT recalculation (DVH_DLCT_) were compared using the Wilcoxon signed-rank test. Because several OAR parameters showed skewed distributions, the median and interquartile range (IQR) were additionally reported alongside the mean ± standard deviation. The patient-wise absolute difference was calculated as |DVH_DLCT_ − DVH_SECT_|, and the relative difference as |DVH_DLCT_ − DVH_SECT_|/DVH_SECT_ × 100%; both were summarized as the mean ± standard deviation. Pairs with a DVH_SECT_ value of zero were excluded only from the relative-difference calculation. Effect sizes for the Wilcoxon signed-rank comparisons were expressed as matched-pairs rank-biserial correlations (r_rb_). Because the comparisons across multiple DVH parameters were exploratory, no formal adjustment for multiple comparisons was applied. Normal-tissue dosimetric parameters were evaluated in the same manner. For the lungs and heart, the mean dose and *V*_5_, *V*_10_, *V*_20_, *V*_30_, and *V*_40_ were assessed, where *V*_x_ was defined as the percentage volume receiving at least *x* Gy(RBE). For the esophagus and spinal cord, *D*_1_, *D*_2_, and mean dose were evaluated. For the ribs, *D*_0.5cc_, *D*_1cc_, *D*_2cc_, as well as *V*_30_ and *V*_40_, were evaluated, where *D*_x_ was defined as the minimum dose received by the hottest *x* cm^3^ of the structure.

## 3. Results

### 3.1. SECT calibration and phantom-based assessment of SPR prediction agreement

**Fig 2:**
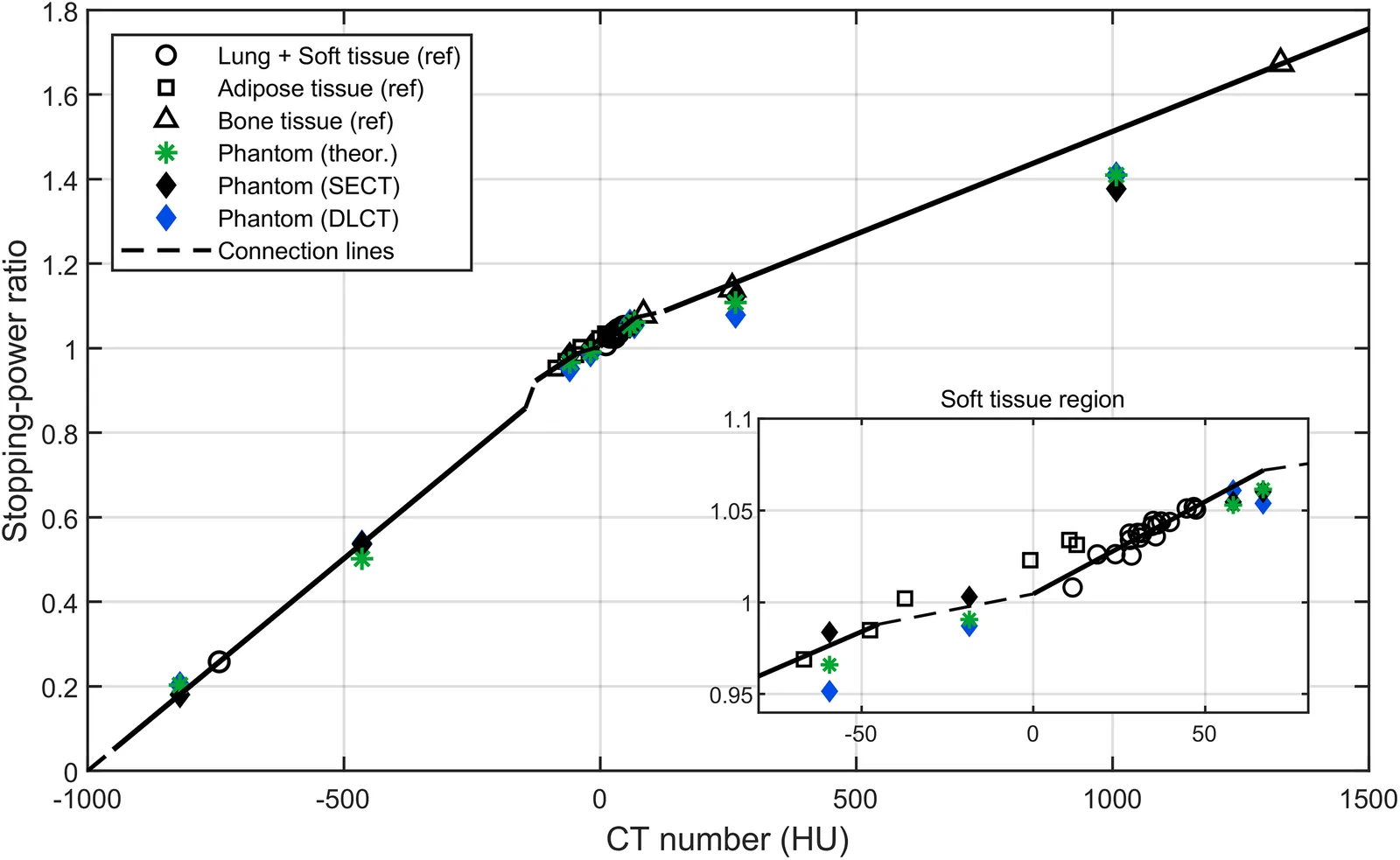
Stoichiometric calibration curve for single-energy computed tomography (SECT)-based SPR prediction. The solid and dashed lines together represent the Hounsfield look-up table used for SECT-based HU-to-SPR conversion. Open markers indicate human reference tissues used for stoichiometric calibration, whereas filled markers indicate the phantom inserts, for which SPR_SECT_, SPR_DLCT_, and SPR_theor_ are plotted along with the calibration curve.

The stoichiometric parameters obtained from the CIRS phantom scan were *k_1_* = 5.00 × 10 ^−^ ⁶ and *k_2_* = 3.41 × 10 ^−^ ^5^. Fig 2 shows the calibration curve generated using these parameters, along with the SPR_theor_, SPR_SECT_, and SPR_DLCT_ values. For the SPR data points, the HU values corresponded to the representative HU values of each insert material, as described in Section 2.4. The measured and calculated HU values of the inserts are provided in S1 File.

**Fig 3:**
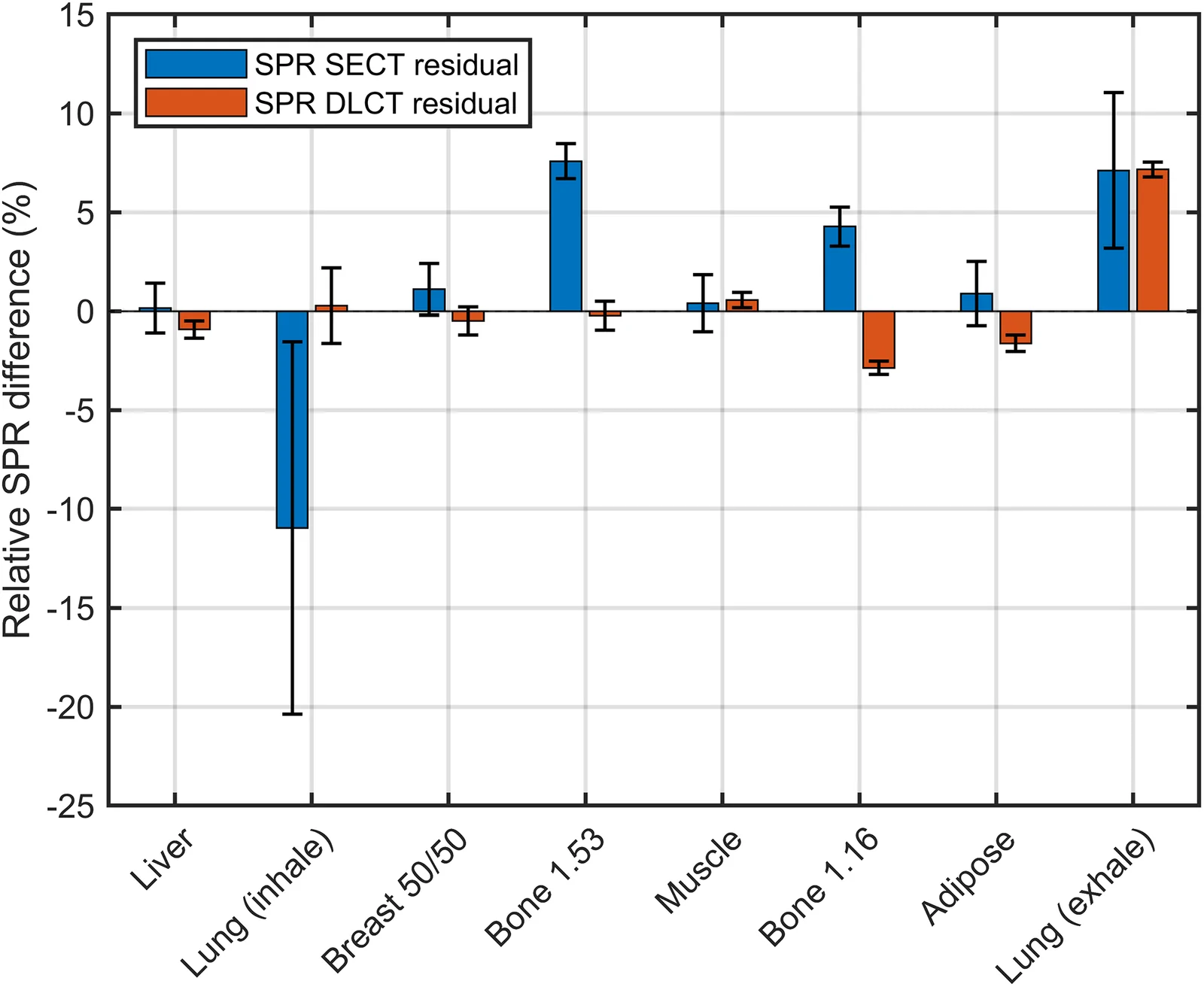
Relative residuals of SECT- and DLCT-based SPR predictions for CIRS phantom plugs. Relative residuals are calculated with respect to the theoretical SPR values of each plug as (SPR_method_ − SPR_theor_)/SPR_theor_ × 100%, where SPR_method_ represents either SPR_SECT_ or SPR_DLCT_. Theoretical SPR values are calculated using the Bethe equation and Bragg additivity rule based on the plug mass density and elemental composition. Error bars represent one standard deviation (1*σ*) of the measured values within the ROI.

Fig 3 shows the relative residuals of SPR_SECT_ and SPR_DLCT_ with respect to SPR_theor_ for each phantom insert material. The root-mean-square error (RMSE) relative to SPR_theor_ was 5.57% for SPR_SECT_ and 2.83% for SPR_DLCT_, indicating better agreement with the theoretical SPR values for DLCT-based SPR prediction. The largest relative deviation was observed for the lung inhale insert in SECT (10.96%) and for the lung exhale insert in DLCT (7.31%). With the lung exhale insert excluded, DLCT showed a mean absolute relative difference of 0.90%. Residuals were lower with DLCT than with SECT, particularly for the lung inhale and high-density bone inserts, whereas similar agreement with the theoretical SPR values was observed for the remaining inserts. The mean relative standard deviation across inserts was also lower for DLCT than for SECT (0.66% vs. 2.61%), indicating more consistent insert-wise SPR estimates with DLCT-based prediction.

### 3.2. Mean SPR for normal tissues

**Fig 4:**
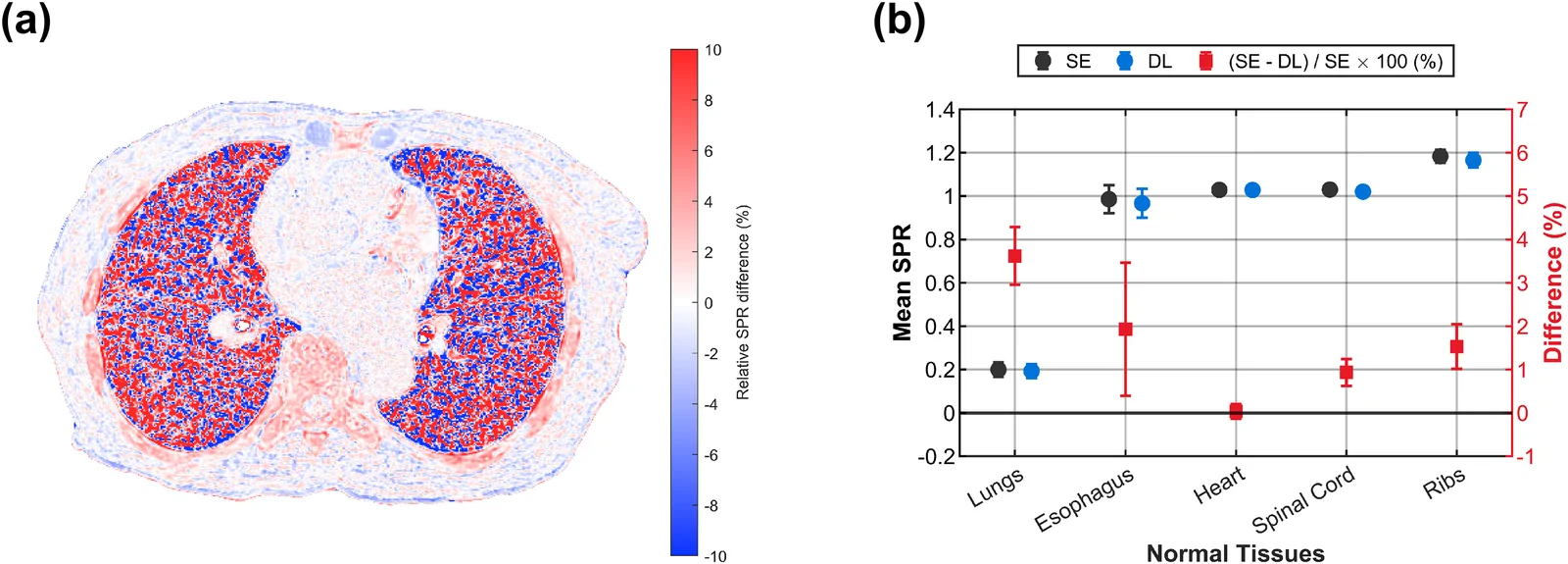
Normal-tissue SPR differences between SECT and DLCT in thoracic patients. (a) Representative axial distribution of the relative difference between SECT- and DLCT-based SPR predictions for a thoracic patient, defined as (SPR_SECT_−SPR_DLCT_)/SPR_SECT_×100%, where SPR_SECT_ and SPR_DLCT_ denote the stopping-power ratios predicted from SECT and DLCT, respectively.(b) Mean SPR values of normal tissues in the 20 thoracic patients derived from SECT (black circles, left axis) and DLCT (blue circles, left axis). The relative difference between SECT- and DLCT-based SPR predictions is first calculated on a per-patient basis for each tissue as (SPR_SECT_−SPR_DLCT_)/SPR_SECT_×100%, and the resulting patient-wise values are summarized as red squares (right axis). Error bars represent standard deviations.

Fig 4(a) presents a representative map of the relative SPR difference between SECT and DLCT. Overall, minimal differences were observed in soft-tissue regions, such as the skin, whereas SECT yielded higher SPR values in bony structures. In lung tissue, the relative differences showed a heterogeneous pattern. Fig 4(b) compares the normal-tissue SPR values predicted from SECT and DLCT in the patient cohort. Across the 20 patients, the mean SPR values differed by 3.62 ± 0.67% for the lungs, 1.93 ± 1.53% for the esophagus, 0.04 ± 0.17% for the heart, 0.93 ± 0.31% for the spinal cord, and 1.53 ± 0.51% for the ribs.

### 3.3. Recalculated range differences by patient

**Fig 5:**
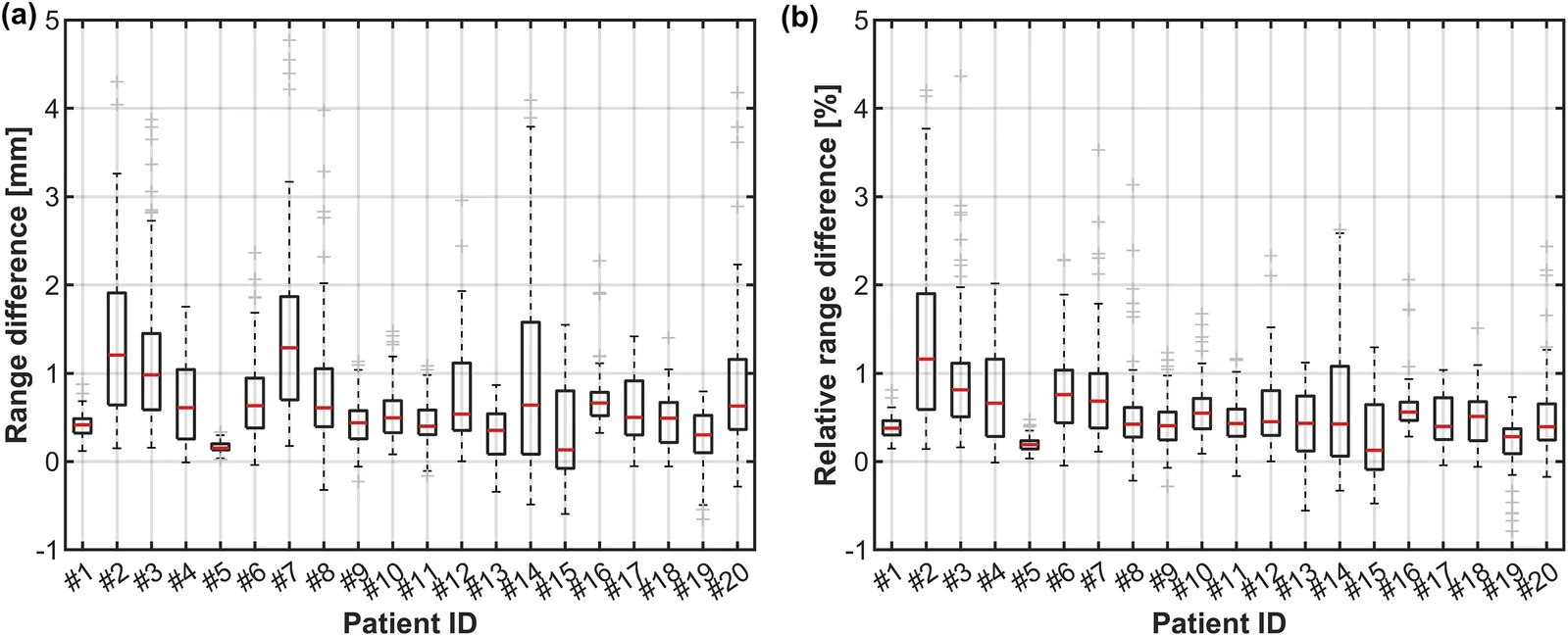
Patient-specific recalculated range differences in the beam’s-eye view, summarized in the form of box-and-whisker plots. Range differences are calculated as the DLCT-based predicted range minus the SECT-based predicted range. (a) Range difference (mm) between the SECT- and DLCT-based predictions. (b) Relative range difference (%), defined as the difference between the SECT- and DLCT-based predicted ranges divided by the SECT-based predicted range. The red line indicates the median, boxes represent the interquartile range, whiskers indicate 1.5 times the interquartile range, and outliers are displayed as gray crosses.

Fig 5 summarizes the patient-specific recalculated range differences in the form of box-and-whisker plots. Across the entire patient cohort, the mean recalculated range difference was 0.71 ± 0.89 mm in Fig 5(a), while the mean relative difference was 0.60 ± 0.67%, as shown in Fig 5(b). For 19 of the 20 patients, the median relative range difference was within the 0%–1% range, indicating overall consistency across the cohort.

### 3.4. Dosimetric comparison between SECT plans and DLCT recalculations

**Fig 6:**
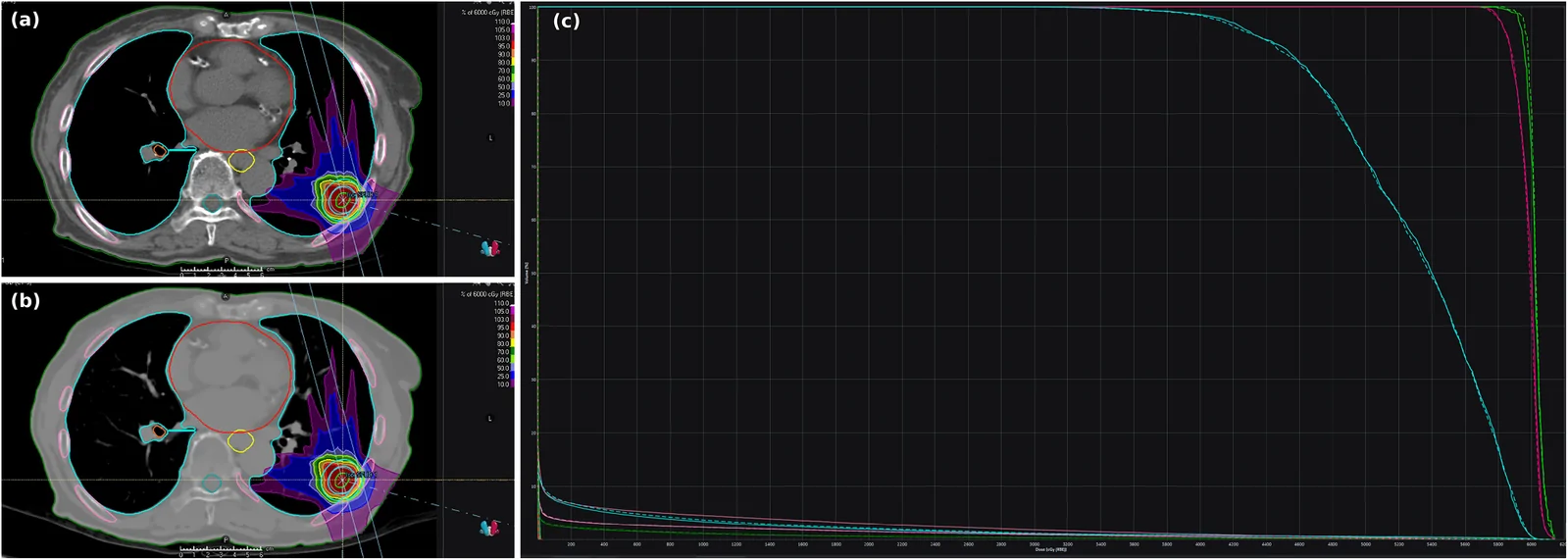
Comparison of SECT-based planning and DLCT-based recalculation in a representative patient. (a) Dose distribution for the SECT-based plan. (b) Dose distribution for the DLCT-based recalculation. (c) Corresponding DVH comparison. Solid and dashed lines indicate the SECT-based plan and DLCT-based recalculation, respectively. Magenta, green, and cyan lines represent the planning target volume (PTV), clinical target volume (CTV), and lung organ-at-risk (OAR) contours, respectively.

Fig 6 presents a representative patient case comparing the SECT-based plan and DLCT-based recalculation. Fig 6(a) and (b) show the dose distributions for the SECT-based plan and DLCT-based recalculation, respectively, with the corresponding DVH comparison shown in Fig 6(c).

Table 2 presents the dosimetric comparison between the SECT-based plans and DLCT recalculations for both the target and OARs.

**Table 2:** Dosimetric comparison of SECT-based treatment plans and DLCT-based recalculations for the target and organs at risk. Values are expressed as mean ± standard deviation and median [interquartile range]. Dose parameters, with mean dose denoted as D_mean_, and their absolute differences are reported in cGy(RBE), whereas absolute differences in V_x_ are expressed in percentage points. Relative differences in dose–volume histogram (DVH) parameters were calculated on a patient-wise basis as |DVH_DLCT_ − DVH_SECT_|/DVH_SECT_ × 100%. Effect sizes are reported as matched-pairs rank-biserial correlations (r_rb_). Statistical comparisons were performed using the Wilcoxon signed-rank test. No adjustment for multiple comparisons was applied.

Target /OAR	DVH parameter	DVH_SECT_ (Mean ± SD; Median [Q1-Q3])	DVH_DLCT_ (Mean ± SD; Median [Q1-Q3])	Absolute difference	Relative difference	*p*-value	r_rb_
PTV	*D*_98_	5732.6 ± 52.50; 5743.58 [5726.21–5758.28]	5729.21 ± 41.96; 5729.76 [5712.79–5764.30]	27.90 ± 27.15	0.49 ± 0.48	0.709	0.095
	*D*_95_	5806.64 ± 39.43; 5810.84 [5800.55–5828.93]	5804.12 ± 32.16; 5808.42 [5788.73–5826.70]	24.28 ± 26.23	0.42 ± 0.46	0.881	−0.038
	*D*_2_	6089.12 ± 25.21; 6080.98 [6075.24–6104.67]	6088.96 ± 25.52; 6085.68 [6073.95–6099.76]	6.22 ± 4.66	0.10 ± 0.08	0.654	−0.114
	D_mean_	5977.90 ± 4.56; 5978.17 [5975.93–5981.12]	5973.62 ± 8.75; 5974.85 [5970.45–5980.63]	7.16 ± 8.07	0.12 ± 0.13	0.167	−0.352
	CI	0.81 ± 0.09; 0.81 [0.73–0.88]	0.80 ± 0.09; 0.80 [0.72–0.88]	0.01 ± 0.01	1.74 ± 1.07	0.147	−0.418
	HI	0.059 ± 0.012; 0.058 [0.052–0.060]	0.060 ± 0.010; 0.060 [0.051–0.063]	0.005 ± 0.004	7.77 ± 6.89	1.000	0.000
CTV	*D*_98_	5926.10 ± 55.06; 5943.04 [5916.46–5952.76]	5932.50 ± 36.59; 5943.42 [5919.00–5955.74]	16.56 ± 21.70	0.28 ± 0.38	0.433	0.200
	*D*_95_	5953.89 ± 30.14; 5958.73 [5946.95–5973.37]	5957.17 ± 19.70; 5958.80 [5948.18–5971.47]	11.78 ± 14.57	0.20 ± 0.25	0.313	0.257
	*D*_2_	6096.26 ± 42.44; 6095.43 [6067.23–6121.26]	6095.25 ± 45.87; 6098.00 [6063.69–6117.76]	7.45 ± 5.99	0.12 ± 0.10	0.391	−0.219
	D_mean_	6016.42 ± 11.87; 6016.89 [6007.10–6023.85]	6016.79 ± 14.29; 6018.69 [6006.04–6028.33]	4.81 ± 4.32	0.08 ± 0.07	0.823	0.057
Lung	*V*_*5*_	6.07 ± 4.70; 4.86 [2.84–7.09]	6.31 ± 4.89; 5.15 [2.95–7.45]	0.24 ± 0.32	4.07 ± 2.59	<0.001	1.000
	*V*_*10*_	4.72 ± 3.67; 3.70 [2.27–5.48]	4.91 ± 3.81; 3.84 [2.34–5.82]	0.19 ± 0.27	4.12 ± 3.15	<0.001	1.000
	*V*_*20*_	3.07 ± 2.29; 2.17 [1.72–3.70]	3.20 ± 2.39; 2.33 [1.75–3.86]	0.13 ± 0.14	4.03 ± 2.69	<0.001	0.914
	*V*_*30*_	2.14 ± 1.67; 1.39 [1.11–2.71]	2.20 ± 1.73; 1.44 [1.14–2.77]	0.07 ± 0.08	3.05 ± 1.86	<0.001	0.876
	*V*_*40*_	1.58 ± 1.26; 1.07 [0.75–2.10]	1.61 ± 1.29; 1.08 [0.75–2.14]	0.04 ± 0.05	2.19 ± 1.38	0.001	0.819
	D_mean_	169.11 ± 125.09; 125.44 [94.22–196.57]	174.51 ± 129.46; 129.27 [96.13–205.20]	5.45 ± 6.38	3.28 ± 2.13	<0.001	0.981
Esophagus	*D*_*1*_	773.79 ± 1938.50; 29.49 [10.85–51.42]	780.66 ± 1949.24; 30.02 [11.15–53.08]	8.88 ± 22.26	3.20 ± 3.17	0.005	0.783
	*D*_*2*_	719.16 ± 1856.12; 24.65 [9.41–48.03]	728.29 ± 1865.94; 25.09 [9.67–49.34]	10.44 ± 25.63	3.20 ± 4.31	0.005	0.783
	D_mean_	167.74 ± 486.63; 3.37 [0.90–7.65]	168.29 ± 484.93; 3.43 [0.92–7.86]	2.33 ± 5.80	2.99 ± 2.06	0.006	0.779
Heart	*V*_*5*_	2.67 ± 6.22; 0.36 [0.00–1.23]	2.72 ± 6.23; 0.41 [0.00–1.27]	0.04 ± 0.06	22.22 ± 30.96	<0.001	1.000
	*V*_*10*_	1.95 ± 4.64; 0.19 [0.00–0.59]	1.99 ± 4.67; 0.22 [0.00–0.65]	0.04 ± 0.05	10.36 ± 12.06	0.002	1.000
	*V*_*20*_	1.46 ± 3.63; 0.06 [0.00–0.32]	1.49 ± 3.67; 0.07 [0.00–0.36]	0.03 ± 0.05	11.59 ± 10.40	0.004	1.000
	*V*_*30*_	1.19 ± 3.03; 0.01 [0.00–0.19]	1.22 ± 3.08; 0.02 [0.00–0.20]	0.03 ± 0.05	16.07 ± 17.40	0.004	1.000
	*V*_*40*_	0.97 ± 2.52; 0.00 [0.00–0.10]	0.99 ± 2.57; 0.00 [0.00–0.11]	0.02 ± 0.05	26.54 ± 45.76	0.004	1.000
	D_mean_	86.13 ± 202.76; 6.63 [3.07–24.84]	87.84 ± 204.86; 7.52 [3.20–26.45]	1.71 ± 2.67	5.75 ± 3.66	<0.001	1.000
Spinal cord	*D*_*1*_	77.15 ± 218.68; 14.56 [2.61–26.59]	77.63 ± 218.25; 14.97 [2.64–27.55]	0.67 ± 0.87	3.01 ± 1.44	0.032	0.692
	*D*_*2*_	67.99 ± 191.37; 13.46 [1.59–24.45]	68.42 ± 191.03; 13.90 [1.60–25.46]	0.56 ± 0.68	2.89 ± 1.40	0.034	0.727
	D_mean_	6.79 ± 18.14; 1.43 [0.11–3.06]	7.00 ± 18.70; 1.48 [0.11–3.17]	0.21 ± 0.56	2.55 ± 1.37	0.003	0.868
Ribs	*V*_*30*_	0.49 ± 0.68; 0.27 [0.10–0.72]	0.48 ± 0.68; 0.24 [0.11–0.72]	0.01 ± 0.01	25.27 ± 74.95	0.124	−0.425
	*V*_*40*_	0.30 ± 0.58; 0.09 [0.00–0.40]	0.30 ± 0.58; 0.08 [0.00–0.39]	0.01 ± 0.01	8.16 ± 10.11	<0.001	−0.983
	*D*_*2cc*_	2591.30 ± 1365.60; 2363.50 [1618.00–3128.00]	2566.10 ± 1373.42; 2395.00 [1666.50–3104.00]	52.80 ± 46.06	4.03 ± 8.24	0.070	−0.486
	*D*_*1cc*_	3369.05 ± 1347.49; 3143.50 [2560.00–4308.50]	3342.75 ± 1328.70; 3127.00 [2598.00–4246.00]	52.40 ± 42.86	1.71 ± 1.43	0.046	−0.510
	*D*_*0.5cc*_	3815.65 ± 1468.68; 3435.50 [2815.00–5157.00]	3818.55 ± 1444.26; 3639.00 [2845.50–5104.00]	72.30 ± 121.99	2.23 ± 3.78	0.057	−0.462

No statistically significant differences were observed in the CTV or PTV dose parameters between the SECT-based plans and DLCT recalculations. In contrast, several OAR metrics showed statistically significant differences, including most parameters for the lung, esophagus, heart, and spinal cord, as well as *V*_40_ and *D*_1cc_ for the ribs. However, the absolute dosimetric variations were minimal—generally within 0.24 percentage points for volumetric parameters and 11 cGy(RBE) for dose parameters—even for metrics with moderate-to-large effect sizes. Comprehensive dosimetric differences and effect sizes are detailed in Table 2.

## 4. Discussion

This study evaluated the impact of DLCT-derived SPR prediction on conventional SECT-based carbon-ion treatment planning for thoracic cancer by comparing phantom-based agreement with theoretical SPR values, patient-specific SPR differences, recalculated range differences, and dosimetric changes.

A phantom-based prediction assessment was performed by comparing SPR_SECT_ and SPR_DLCT_ with theoretical SPR values for the CIRS 062M inserts. Overall, DLCT-based SPR prediction showed a better agreement with the theoretical SPR values than SECT-based SPR prediction, particularly for the lung inhale and bone inserts. The improved agreement is reasonable because DLCT-based prediction utilizes both RED and EAN to derive the mean excitation energy, which is more directly related to particle stopping power than HU alone. The use of these DLCT-derived quantities is also consistent with a previous report showing accurate RED and EAN estimation using DLCT [36].

The overestimation of SPR_SECT_ for the bone inserts is likely attributable to compositional differences between the phantom inserts and the human reference tissues used for stoichiometric calibration. For example, the Bone 1.53 insert had a measured HU substantially higher than the HU corresponding to SPR_theor_ on the calibration curve, although its SPR was only 1.6% higher than that of the skeleton-ribs (10th) reference tissue, compared with 37% and 9.2% higher HU and EAN values, respectively (see S1 File). This suggests that kilovoltage CT calibration may be affected by EAN-dependent photon attenuation and associated beam-hardening effects in high-EAN materials [37,38]. Therefore, part of the larger SECT residual and RMSE may reflect the mismatch between the plastic phantom inserts and the human reference tissues used for calibration, rather than an intrinsic limitation of SECT-based SPR prediction. Accordingly, the RMSE values of 5.57% for SECT and 2.83% for DLCT should be interpreted as phantom-specific agreement with the calculated reference SPR values. The relatively large residual for the lung exhale insert, which was also observed with the DLCT-based prediction, likely reflects insert-specific uncertainty related to the structural heterogeneity of lung-equivalent materials, rather than limitations of the HLUT alone [25, 39].

In the patient analysis, the differences in the SPR predictions between SECT and DLCT were tissue dependent. Differences were minimal in soft-tissue-dominant regions, including the heart and spinal cord, whereas larger mean differences were observed in the esophagus, ribs, and lungs. The variability in the SPR differences within the esophagus may be related to patient-specific differences in the air content, while the relatively large percentage difference in the lungs may be partly amplified by the low absolute SPR of lung tissue. These findings suggest that the SPR differences between SECT and DLCT are partly related to differences between the reference tissue compositions used for stoichiometric calibration and the actual patient tissues. In bony tissues, the consistently higher SECT-based SPR values, as shown in Fig 4(a), correspond to the SPR overestimation observed for high-EAN bone inserts in the phantom assessment, suggesting that EAN-related compositional effects may also be present in patient anatomy. Because patient-specific elemental compositions were unavailable, this interpretation remains limited, and further validation using biological tissues with known compositions will be required.

SECT-based plans and DLCT-based recalculations were evaluated in terms of range differences. Across all patients, the mean relative range difference was 0.6%. For a carbon-ion beam with a range of 15 cm, this corresponds to a DLCT-based calculation predicting that the beam would travel approximately 0.9 mm further for the same plan. The mean relative range difference between the two image sets was comparable to previously reported values [14].

Finally, a dosimetric comparison between the SECT-based plans and DLCT recalculations was performed for the target and OARs. No significant differences were observed in the CTV or PTV dose parameters. Given that most range differences reported in Section 3.3 were less than 4 mm, the absence of significant CTV and PTV dose differences is consistent with the use of a 5-mm PTV margin. Within this static image-based analysis, the applied margin appeared sufficient to maintain target coverage against method-related range differences. In contrast, several OAR metrics showed statistically significant but small differences, likely reflecting the cumulative impact of SPR deviations along the beam path. While the moderate-to-large effect sizes indicate a systematic difference between the two calculation methods, the absolute dosimetric variations remained numerically small. Furthermore, the comparatively large relative differences observed in certain low-volume heart metrics were primarily driven by their near-zero baseline doses.

For the ribs, V_40_ and D_1cc_ showed statistically significant differences, with mean absolute differences of 0.01 percentage points and 52.40 cGy(RBE), respectively. High-dose rib dose–volume parameters have been reported as predictors of radiation-induced rib fracture following thoracic irradiation [40,41]. For the other OARs, DLCT-based recalculation may be more important for central thoracic or mediastinal tumors, where critical structures, such as the esophagus, are closer to the high-dose region [42,43].

The main focus of this study was to evaluate the impact of DLCT-based SPR prediction on current SECT-based carbon-ion treatment planning for thoracic cancer. Although DECT- or spectral CT-based SPR predictions have been investigated for several anatomical sites, including the brain and head-and-neck, its impact on carbon-ion treatment planning for thoracic cancer remains underexplored. In particular, the effect of applying DLCT-derived SPR information to the same SECT-based treatment plan has not been fully evaluated [19]. Imaging-related uncertainty is a major source of the overall uncertainty in thoracic cancer treatment; however, within this context, the uncertainties associated with DLCT-based SPR prediction have received comparatively less attention. Although the present study did not incorporate respiratory motion, it provides a baseline estimate of the impact of DLCT-derived SPR information on thoracic carbon-ion treatment planning. Further motion-resolved studies are required to evaluate whether patient-specific SPR assessment can support margin reduction in thoracic applications. Because this study included various thoracic cancer types, inter-patient variability in DVH differences was expected and likely reflects differences in tumor location, target size, beam arrangement, and anatomical composition along the beam path. Because multiple DVH endpoints were evaluated without formal adjustment for multiple comparisons, individual p-values should be interpreted cautiously. Accordingly, the statistical findings were considered together with the absolute and relative differences and effect sizes. Further studies with larger cohorts focused on specific thoracic tumor sites are required to clarify the clinical relevance of these dosimetric differences.

The present study has several limitations. First, only a single EAN-to-ln *I* conversion function was used for the DLCT-based SPR prediction. Consequently, uncertainties associated with the selection of alternative conversion models were not evaluated. Second, verifying the SPR_DLCT_ prediction would ideally require an independent phantom that differs from the calibration phantom. Because the same CIRS 062M phantom was used to derive the SECT HLUT and to perform the insert-based comparison, the phantom dataset was not independent of the SECT calibration procedure. Accordingly, the observed agreement with theoretical insert SPR values should be interpreted as a phantom-based prediction assessment rather than an independent validation of DLCT-derived SPR accuracy. Independent validation using a separate phantom or biological tissues with experimentally measured reference SPR values is required. Third, because the plan recalculations were performed using the clinical pencil-beam algorithm and modified MKM implemented in RayStation, the present comparison primarily reflects the effect of changes in SPR on water-equivalent path length and the resulting range and RBE-weighted dose distributions. Element-specific nuclear fragmentation and composition-dependent RBE effects were not explicitly evaluated; assessment of these effects would require a Monte Carlo framework incorporating elemental composition. Fourth, the analysis did not incorporate 4D imaging, which represents a significant limitation for thoracic applications. Respiratory motion can alter the beam-path water-equivalent path length, with patient-based 4D CT studies reporting phase-dependent changes on the order of approximately 1–4 mm [44,45]. These variations are larger than the mean DLCT–SECT recalculated range difference observed in the present study, suggesting that the SPR-related contribution observed in the SECT–DLCT recalculation may be subdominant relative to respiratory range uncertainties in thoracic cancer. Nevertheless, with continued advances in respiratory-gated particle therapy and 4D dual-energy CT, the magnitude reported here may provide a useful reference for the SPR-related component of range uncertainty when considering range margins. Further studies using 4D spectral CT with motion-resolved quantitative assessment are therefore required to clarify the clinical relevance of DLCT-based SPR prediction in thoracic carbon-ion therapy.

## 5. Conclusions

This study investigated the impact of DLCT-derived SPR prediction on thoracic carbon-ion treatment planning by comparing it with the conventional SECT-based workflow. DLCT showed better agreement with theoretical SPR values in a phantom-based prediction assessment and demonstrated tissue-dependent SPR differences in patient images, particularly in the lung and other heterogeneous thoracic regions. Recalculation of SECT-based plans on DLCT-derived SPR maps resulted in modest but systematic range differences, with limited impact on target coverage but observable differences in selected OAR dose metrics. These findings should be interpreted as a static, image-based baseline assessment of the impact of DLCT-derived SPR information rather than as a clinically generalizable evaluation across thoracic tumor sites. Further site-specific and motion-resolved studies are required to establish the clinical relevance of DLCT-based SPR workflows in thoracic carbon-ion therapy.

## Supporting information

S1 File Supplementary material. Patient-level clinical characteristics, treatment sites, lesion locations, target volumes, and beam angles; SECT- and DLCT-based calibration procedures and reference data; and the line-dose profile extraction procedure used for range analysis.
(DOCX)

S2 File MATLAB analysis code. De-identified MATLAB scripts used for DLCT-derived SPR generation, SECT calibration and phantom-based SPR assessment, range analysis, and DVH and statistical analyses, together with README documentation.
(ZIP)
