# Supplementary material for: Impact of dual-layer spectral CT-derived stopping power ratio on single-energy CT-based carbon-ion treatment planning for thoracic cancer
Source: PLoS One. 2026 Sep 15;21(9):e0358184. doi: 10.1371/journal.pone.0358184 (PMC13577390; doi:10.1371/journal.pone.0358184)
Supplement: Supplementary file 1 [file pone.0358184.s001.docx]

S1. Patient-level clinical characteristics, treatment sites, and beam angles

| Patient # | Cancer type | TNM stage^a^ | CTV (cm^3^) | Site | Lesion location^b^ | Beam angle(°) | | | |  |
| --- | --- | --- | --- | --- | --- | --- | --- | --- | --- | --- |
| 1 | ADC | cT1N0 | 9.09 | RUL | Peripheral | 225 | 240 | 270 | 285 | |
| 2 | SqCC | cT1bN0M0 | 7.00 | LLL | Peripheral | 105 | 120 | 180 | 195 | |
| 3 | Mets | - | 1.33 | LUL | Peripheral | 90 | 105 | 165 | 180 | |
| 4 | SqCC | Masaoka Ⅳ | 122.76 | Thymus | Central | 0 | 30 | 210 | 330 | |
| 5 | ADC | cT1bN0 | 5.60 | RLL | Peripheral | 150 | 165 | 180 | 195 | |
| 6 | ADC | cT2aN0M0 | 0.80 | RLL | Peripheral | 165 | 180 | 195 | 210 | |
| 7 | ADC | pT4xN0M0 | 1.60 | RUL | Central | 165 | 180 | 195 | 210 | |
| 8 | Oligomets | - | 7.56 | RUL | Central | 0 | 30 | 270 | 300 | |
| 9 | Mets | - | 5.13 | Interlobar LN | Central | 60 | 75 | 135 | 150 | |
| 10 | ADC | pT2aN0M0 | 3.98 | Subaortic LN | Central | 0 | 15 | 30 | 45 | |
| 11 | Mets | - | 3.36 | RUL | Central | 15 | 165 | 195 | 345 | |
| 12 | Recur | - | 2.21 | LLL | Peripheral | 135 | 150 | 180 | 195 | |
| 13 | ADC | cT2N2M0 | 1.88 | RUL | Central | 0 | 315 | 330 | 345 | |
| 14 | -^c^ | - | 46.46 | LLL | Peripheral | 90 | 120 | 150 | 180 | |
| 15 | Mets | - | 3.52 | LUL | Peripheral | 30 | 45 | 150 | 180 | |
| 16 | NSCLC | T4N2M0 | 11.96 | LUL | Central | 0 | 15 | 30 | 45 | |
| 17 | Mets | - | 6.08 | LLL | Central | 135 | 150 | 180 | 195 | |
| 18 | ADC | pT2bN2M0 | 55.21 | LUL | Central | 75 | 90 | 120 | 135 | |
| 19 | ADC | pT1bN0M0 | 8.00 | RUL | Peripheral | 0 | 270 | 300 | 330 | |
| 20 | ADC | cT1cN0M0 | 11.54 | LUL | Central | 15 | 165 | 195 | 345 | |

* Abbreviations: #, number; LN, lymph node; ADC, adenocarcinoma; SqCC, squamous cell carcinoma; NSCLC, non-small cell lung cancer, subtype not specified; Mets, metastatic disease; Oligomets, oligometastatic disease

* a: cT and pT denote clinical and pathological T staging, respectively. Thymic carcinoma was staged using the Masaoka system.

* b: Central: lesions located toward the mediastinal/hilar side or adjacent to central thoracic structures such as the heart, Peripheral: lesions located toward the outer lung or chest wall

* c: This patient had no biopsy confirmation.

S2. Calibration process for SECT and DLCT

**DLCT**

Piecewise linear and fifth-order polynomial representation of the relationship between effective atomic number and the natural logarithm of mean excitation energy

Figure S1. Relationship between effective atomic number (Z) and mean excitation energy (I).^1,2^

[Result]

Z < Z_min_: e_1_Z+e_2_

Z_min_ < Z < Z_max_: e_3_Z^5^+e_4_Z^4^+e_5_Z^3^+e_6_Z^2^+e_7_Z^1^+e_8_

Z > Z_max_: e_9_Z+e_10_

[e1 e2 e3 e4 e5 e6 e7 e8 e9 e10]

= [10.8450 -3.8993 -0.0150 0.7006 -12.5478 106.9144 -425.2621 682.5467 12.1258 -48.7141]

**SECT**

Figure S2. Arrangement of CIRS 062M phantom inserts on the conventional HU image. The displayed image corresponds to the SECT-equivalent CT image used for HU measurement and HLUT generation. Insert labels indicate the tissue-equivalent materials used for phantom-based SPR assessment.

Figure S3. Comparison of measured and calculated Hounsfield units (HU) for CIRS phantom inserts. Each point represents an individual insert measurement, with repeated measurements denoted by labels (1) and (2). The dashed line indicates perfect agreement between the measured and calculated HU values. The inserts cover a range of tissue-equivalent materials, including lung, adipose, breast, muscle, liver, and bone-equivalent materials.

Table S1. Elemental compositions and measured/calculated HU values for CIRS 062M inserts.^3^

| Insert  label | H  (%) | C  (%) | N  (%) | O  (%) | Cl  (%) | Ca  (%) | P  (%) | Ba  (%) | ρ  (g/cm^3^) | EAN | SPR | HU (Measured) | HU (calculated) |
| --- | --- | --- | --- | --- | --- | --- | --- | --- | --- | --- | --- | --- | --- |
| Liver | 9 | 69.4 | 2.1 | 17.2 | 0.1 | 2.2 | 0 | 0 | 1.07 | 7.26 | 1.062 | 66.86 | 37.75 |
| Lung (inhale) | 8.8 | 67.5 | 3.5 | 18.6 | 1.6 | 0 | 0 | 0 | 0.205 | 6.69 | 0.203 | -819.90 | -806.16 |
| Breast | 9.6 | 70.3 | 1.9 | 17.1 | 0.2 | 0.9 | 0 | 0 | 0.99 | 6.65 | 0.991 | -18.65 | -48.05 |
| Bone 1.53 | 5.7 | 40.8 | 1 | 25.9 | 0.1 | 17.9 | 8.3 | 0.3 | 1.53 | 12.96 | 1.41 | 1007.14 | 1019.00 |
| Muscle | 9.1 | 69.7 | 2.1 | 16.8 | 0.1 | 2.2 | 0 | 0 | 1.06 | 7.25 | 1.053 | 58.16 | 29.65 |
| Bone 1.16 | 7. | 56.3 | 2 | 22.7 | 0.2 | 8.5 | 3.3 | 0 | 1.16 | 9.83 | 1.108 | 264.28 | 210.33 |
| Adipose | 10 | 71.3 | 1.8 | 16.4 | 0.2 | 0.3 | 0 | 0 | 0.96 | 6.30 | 0.966 | -59.33 | -79.26 |
| Lung (exhale) | 8.9 | 66 | 2.4 | 20.4 | 0.6 | 1.7 | 0 | 0 | 0.507 | 7.23 | 0.502 | -464.72 | -510.43 |

Table S2. Elemental composition of human reference tissue ^4,5^

|  | H (%) | C (%) | N (%) | O (%) | Na (%) | Mg (%) | P (%) | S (%) | Cl (%) | K (%) | Fe (%) | Ca (%) | I (%) | ρ  (g/cm^3^) | EAN | SPR | HU  (calculated) |
| --- | --- | --- | --- | --- | --- | --- | --- | --- | --- | --- | --- | --- | --- | --- | --- | --- | --- |
| Adipose tissue | 11.4 | 59.8 | 0.7 | 27.8 | 0.1 | 0 | 0 | 0.1 | 0.1 | 0 | 0 | 0 | 0 | 0.95 | 6.33 | 0.969 | -66.78 |
| Blood | 10.2 | 11 | 3.3 | 74.5 | 0.1 | 0 | 0.1 | 0.2 | 0.3 | 0.2 | 0.1 | 0 | 0 | 1.06 | 7.53 | 1.050 | 47.34 |
| Brain | 10.7 | 14.5 | 2.2 | 71.2 | 0.2 | 0 | 0.4 | 0.2 | 0.3 | 0.3 | 0 | 0 | 0 | 1.04 | 7.45 | 1.037 | 34.02 |
| Breast | 10.6 | 33.2 | 3 | 52.7 | 0.1 | 0 | 0.1 | 0.2 | 0.1 | 0 | 0 | 0 | 0 | 1.02 | 6.93 | 1.023 | -0.91 |
| Cell nucleus | 10.6 | 9 | 3.2 | 74.2 | 0 | 0 | 2.6 | 0.4 | 0 | 0 | 0 | 0 | 0 | 1.00 | 7.72 | 0.994 | -0.88 |
| Eye lens | 9.6 | 19.5 | 5.7 | 64.6 | 0.1 | 0 | 0.1 | 0.3 | 0.1 | 0 | 0 | 0 | 0 | 1.07 | 7.22 | 1.056 | 35.84 |
| GI tract | 10.6 | 11.5 | 2.2 | 75.1 | 0.1 | 0 | 0.1 | 0.1 | 0.2 | 0.1 | 0 | 0 | 0 | 1.03 | 7.35 | 1.026 | 18.59 |
| Heart | 10.3 | 12.1 | 3.2 | 73.4 | 0.1 | 0 | 0.1 | 0.2 | 0.3 | 0.2 | 0.1 | 0 | 0 | 1.03 | 7.51 | 1.022 | 19.02 |
| Kidney | 10.3 | 13.2 | 3 | 72.4 | 0.2 | 0 | 0.2 | 0.2 | 0.2 | 0.2 | 0 | 0.1 | 0 | 1.05 | 7.44 | 1.042 | 36.07 |
| Liver | 10.2 | 13.9 | 3 | 71.6 | 0.2 | 0 | 0.3 | 0.3 | 0.2 | 0.3 | 0 | 0 | 0 | 1.06 | 7.46 | 1.051 | 44.63 |
| Lung(deflated) | 10.3 | 10.5 | 3.1 | 74.9 | 0.2 | 0 | 0.2 | 0.3 | 0.3 | 0.2 | 0 | 0 | 0 | 1.05 | 7.49 | 1.042 | 36.93 |
| Lymph | 10.8 | 4.1 | 1.1 | 83.2 | 0.3 | 0 | 0 | 0.1 | 0.4 | 0 | 0 | 0 | 0 | 1.02 | 7.48 | 1.022 | 21.03 |
| Muscle | 10.2 | 14.3 | 3.4 | 71 | 0.1 | 0 | 0.2 | 0.3 | 0.1 | 0.4 | 0 | 0 | 0 | 1.05 | 7.45 | 1.042 | 34.52 |
| Ovary | 10.5 | 9.3 | 2.4 | 76.8 | 0.2 | 0 | 0.2 | 0.2 | 0.2 | 0.2 | 0 | 0 | 0 | 1.05 | 7.46 | 1.044 | 39.71 |
| Pancreas | 10.6 | 16.9 | 2.2 | 69.4 | 0.2 | 0 | 0.2 | 0.1 | 0.2 | 0.2 | 0 | 0 | 0 | 1.04 | 7.32 | 1.037 | 28.01 |
| Cartilage | 9.6 | 9.9 | 2.2 | 74.4 | 0.5 | 0 | 2.2 | 0.9 | 0.3 | 0 | 0 | 0 | 0 | 1.10 | 7.88 | 1.079 | 84.54 |
| Red marrow | 10.5 | 41.4 | 3.4 | 43.9 | 0 | 0 | 0.1 | 0.2 | 0.2 | 0.2 | 0.1 | 0 | 0 | 1.03 | 6.96 | 1.034 | 10.47 |
| Spongiosa | 8.5 | 40.4 | 2.8 | 36.7 | 0.1 | 0.1 | 3.4 | 0.2 | 0.2 | 0.1 | 0.1 | 7.4 | 0 | 1.18 | 9.76 | 1.140 | 257.72 |
| Yellow marrow | 11.5 | 64.4 | 0.7 | 23.1 | 0.1 | 0 | 0 | 0.1 | 0.1 | 0 | 0 | 0 | 0 | 0.98 | 6.22 | 1.002 | -37.37 |
| Skin | 10 | 20.4 | 4.2 | 64.5 | 0.2 | 0 | 0.1 | 0.2 | 0.3 | 0.1 | 0 | 0 | 0 | 1.09 | 7.26 | 1.081 | 64.44 |
| Spleen | 10.3 | 11.3 | 3.2 | 74.1 | 0.1 | 0 | 0.3 | 0.2 | 0.2 | 0.3 | 0 | 0 | 0 | 1.06 | 7.48 | 1.052 | 46.68 |
| Testis | 10.6 | 9.9 | 2 | 76.6 | 0.2 | 0 | 0.1 | 0.2 | 0.2 | 0.2 | 0 | 0 | 0 | 1.04 | 7.44 | 1.035 | 30.98 |
| Thyroid | 10.4 | 11.9 | 2.4 | 74.5 | 0.2 | 0 | 0.1 | 0.1 | 0.2 | 0.1 | 0 | 0 | 0.1 | 1.05 | 7.94 | 1.043 | 79.31 |
| Skeleton-corticalbone | 3.4 | 15.5 | 4.2 | 43.5 | 0.1 | 0.2 | 10.3 | 0.3 | 0 | 0 | 0 | 22.5 | 0 | 1.92 | 13.23 | 1.674 | 1327.79 |
| Skeleton-cranium | 5 | 21.2 | 4 | 43.5 | 0.1 | 0.2 | 8.1 | 0.3 | 0 | 0 | 0 | 17.6 | 0 | 1.61 | 12.27 | 1.451 | 876.30 |
| Skeleton-femur | 7 | 34.5 | 2.8 | 36.8 | 0.1 | 0.1 | 5.5 | 0.2 | 0.1 | 0 | 0 | 12.9 | 0 | 1.33 | 11.12 | 1.247 | 492.41 |
| Skeleton-humerus | 6 | 31.4 | 3.1 | 36.9 | 0.1 | 0.1 | 7 | 0.2 | 0 | 0 | 0 | 15.2 | 0 | 1.46 | 11.69 | 1.344 | 667.69 |
| Skeleton-mandible | 4.6 | 19.9 | 4.1 | 43.5 | 0.1 | 0.2 | 8.6 | 0.3 | 0 | 0 | 0 | 18.7 | 0 | 1.68 | 12.50 | 1.502 | 974.67 |
| Skeleton-ribs(2nd,6th) | 6.4 | 26.3 | 3.9 | 43.6 | 0.1 | 0.1 | 6 | 0.3 | 0.1 | 0.1 | 0 | 13.1 | 0 | 1.41 | 11.30 | 1.308 | 579.83 |
| Skeleton-ribs(10th) | 5.6 | 23.5 | 4 | 43.4 | 0.1 | 0.1 | 7.2 | 0.3 | 0.1 | 0.1 | 0 | 15.6 | 0 | 1.52 | 11.87 | 1.387 | 742.21 |
| Skeleton-sacrum | 7.4 | 30.2 | 3.7 | 43.8 | 0 | 0.1 | 4.5 | 0.2 | 0.1 | 0.1 | 0.1 | 9.8 | 0 | 1.29 | 10.51 | 1.221 | 403.06 |
| Skeleton-spongiosa | 8.5 | 40.4 | 2.8 | 36.7 | 0.1 | 0.1 | 3.4 | 0.2 | 0.2 | 0.1 | 0.1 | 7.4 | 0 | 1.18 | 9.76 | 1.140 | 257.72 |
| Skeleton-vertebral (C4) | 6.3 | 26.1 | 3.9 | 43.6 | 0.1 | 0.1 | 6.1 | 0.3 | 0.1 | 0.1 | 0 | 13.3 | 0 | 1.42 | 11.35 | 1.314 | 593.08 |
| Skeleton-vertebral (D6,L3) | 7 | 28.7 | 3.8 | 43.7 | 0 | 0.1 | 5.1 | 0.2 | 0.1 | 0.1 | 0.1 | 11.1 | 0 | 1.33 | 10.84 | 1.249 | 465.19 |

S3. Line dose profile.

Figure S4. Illustration of line-dose profile extraction along the incident beam direction for patient #5 listed in the patient-level characteristics section of this file. For each beam direction, three line-dose profiles were defined to pass through the PTV on the axial dose image, with each profile aligned parallel to the corresponding beam direction. Although a total of 12 line profiles were defined across the four beam directions, only the three profiles corresponding to the 180° beam direction are shown in this representative illustration. The same three profile positions were repeated over five consecutive slices, resulting in 15 extracted profiles per beam.

**Reference**

1.	Bourque AE, Carrier JF, Bouchard H. A stoichiometric calibration method for dual energy computed tomography. *Phys Med Biol*. Apr 21 2014;59(8):2059–88. doi:10.1088/0031-9155/59/8/2059

2.	Schneider W, Bortfeld T, Schlegel W. Correlation between CT numbers and tissue parameters needed for Monte Carlo simulations of clinical dose distributions. *Phys Med Biol*. Feb 2000;45(2):459–78. doi:10.1088/0031-9155/45/2/314

3.	Almeida IP, Schyns L, Vaniqui A, et al. Monte Carlo proton dose calculations using a radiotherapy specific dual-energy CT scanner for tissue segmentation and range assessment. *Phys Med Biol*. May 29 2018;63(11):115008. doi:10.1088/1361-6560/aabb60

4.	Schneider U, Pedroni E, Lomax A. The calibration of CT Hounsfield units for radiotherapy treatment planning. *Phys Med Biol*. Jan 1996;41(1):111–24. doi:10.1088/0031-9155/41/1/009

5.	Woodard HQ, White DR. The composition of body tissues. *Br J Radiol*. Dec 1986;59(708):1209–18. doi:10.1259/0007-1285-59-708-1209
